# Supplementary material for: EnzML: multi-label prediction of enzyme classes using InterPro signatures
Source: BMC Bioinformatics. 2012 Apr 25;13:61. doi: 10.1186/1471-2105-13-61 (PMC3483700; doi:10.1186/1471-2105-13-61)
Supplement: Addtional file 5 — The Java code to format the data files, evaluate and predict. The file enzml_java_code.tar.gz contains the Java code used to format database data to ARFF and XML formats, to execute cross and train-test (jackknife) evaluations and to record evaluation results to database. More information is included in the readme.txt file and the Javadoc files. The code can be used with a MySQL database. To use a different database software, other JDBC drivers might be required. [file 1471-2105-13-61-S5.gz › java_code/utils/doc/uk/ac/ed/inf/utils/database/Table.html]

Table


---


|  |  |  |
| --- | --- | --- |
| **Overview** \| **Package** \| **Class** \| **Use** \| **Tree** \| **Deprecated** \| **Index** \| **Help** | |  |
| **PREV CLASS**   **NEXT CLASS** | **FRAMES**    **NO FRAMES**     **All Classes** |  |
| SUMMARY: NESTED \| FIELD \| CONSTR \| METHOD | DETAIL: FIELD \| CONSTR \| METHOD |  |


---


## uk.ac.ed.inf.utils.database Class Table

```
java.lang.Object
  uk.ac.ed.inf.utils.database.Table
```

---

``` public class Table extends java.lang.Object ```

An SQL database table definition (table name + columns)

**Version:**
:   10 Jun 2010

**Author:**
:   Luna De Ferrari luna.deferrari-at-ed.ac.uk

---

**Constructor Summary**

`Table(java.lang.String tableName,
java.util.Vector<TableColumn> columns)`
  
          List of primary key fields (starting from 0)


| **Method Summary** | |
| --- | --- |
| `java.util.Vector<java.lang.String>` | `getColumnNames()`             Gets column names for the table |
| `java.util.Vector<TableColumn>` | `getColumns()`             Get the `TableColumn` list |
| `java.lang.String` | `getTableName()`             Get the table name |
| `boolean` | `hasKey()`             Gets the "has primary key" status for the table |

**Methods inherited from class java.lang.Object**

`equals, getClass, hashCode, notify, notifyAll, toString, wait, wait, wait`

**Constructor Detail**

### Table

```
public Table(java.lang.String tableName,
             java.util.Vector<TableColumn> columns)
```

:   List of primary key fields (starting from 0)


**Method Detail**

### getColumnNames

```
public java.util.Vector<java.lang.String> getColumnNames()
```

:   Gets column names for the table

    :   **Returns:**: the column names

---


### getColumns

```
public java.util.Vector<TableColumn> getColumns()
```

:   Get the `TableColumn` list

    :   **Returns:**: the `TableColumn` list

---


### getTableName

```
public java.lang.String getTableName()
```

:   Get the table name

    :   **Returns:**: a string with the table name

---


### hasKey

```
public boolean hasKey()
```

:   Gets the "has primary key" status for the table


---


|  |  |  |
| --- | --- | --- |
| **Overview** \| **Package** \| **Class** \| **Use** \| **Tree** \| **Deprecated** \| **Index** \| **Help** | |  |
| **PREV CLASS**   **NEXT CLASS** | **FRAMES**    **NO FRAMES**     **All Classes** |  |
| SUMMARY: NESTED \| FIELD \| CONSTR \| METHOD | DETAIL: FIELD \| CONSTR \| METHOD |  |


---
